# Marine bacteria harbor the sulfonamide resistance gene *sul4* without mobile genetic elements

**DOI:** 10.3389/fmicb.2023.1230548

**Published:** 2023-09-14

**Authors:** Suzune Shindoh, Aya Kadoya, Reo Kanechi, Kozo Watanabe, Satoru Suzuki

**Affiliations:** ^1^Center for Marine Environmental Studies, Ehime University, Matsuyama, Japan; ^2^Graduate School of Science and Engineering, Ehime University, Matsuyama, Japan

**Keywords:** *sul4*, sulfonamide resistance, dihydropteroate synthase, marine bacteria, α-proteobacteria

## Abstract

Marine bacteria are possible reservoirs of antibiotic-resistance genes (ARGs) originating not only from clinical and terrestrial hot spots but also from the marine environment. We report here for the first time a higher rate of the sulfonamide-resistance gene *sul4* in marine bacterial isolates compared with other *sul* genes. Among four sulfonamide-resistance genes (*sul1*, *sul2*, *sul3*, and *sul4*), *sul4* was most abundant (45%) in 74 sulfonamide-resistant marine isolates by PCR screening. The order of abundance was *sul4* (33 isolates) *>sul2* (6 isolates) *>sul3* (5 isolates) *>sul1* (1 isolate). Whole-genome sequencing of 23 isolates of *sul4*-expressing α- and γ-proteobacteria and bacilli revealed that *sul4* was not accompanied by known mobile genetic elements. This suggests that *sul4* in these marine isolates is clonally transferred and not horizontally transferable. Folate metabolism genes formed a cluster with *sul4*, suggesting that the cluster area plays a role in folate metabolism, at which *sul4* functions as a dihydropteroate synthase. Thus, *sul4* might be expressed in marine species and function in folate synthesis, but it is not a transferable ARG.

## 1. Introduction

Antibiotic-resistant bacteria (ARB) appeared almost immediately after the use of antibiotics began and now represents a major global health issue in human and animal chemotherapy. ARB develop via conservation of DNA mutations and the acquisition of antibiotic-resistance genes (ARGs) through horizontal gene transfer (HGT) (Levy and Marchall, 2004). HGT is thought to occur between bacteria via intra- and inter-species mechanisms. The One Health concept (G7 Presidency, 2015) involves a humans-animals-environment linkage in which HGT should be considered in relation to not only clinical but also environmental bacteria. ARGs could enter humans and animals from natural environmental bacteria.

Sulfonamides are synthetic antimicrobial agents and the first drugs used for chemotherapy of bacterial and protozoan infections in 1935 (Bickel, 1988). In developed countries, the use of sulfonamides in humans has been replaced by more-advanced antibiotics, but these drugs are still widely used in humans and animals in developing countries (Shimizu et al., 2013). During the long history of sulfonamides, many analogues and combination use with trimethoprim have been developed to overcome the development of resistant bacteria. Bacteria resistant to sulfonamides can be detected all over the world, and selective pressure (Na et al., 2014) and the stability of the compounds (Sukul and Spiteller, 2006; Zhang et al., 2013; Matsuura et al., 2021) have played a role in the development and dissemination of sulfonamide-resistant bacteria in the environment.

The main mechanism of sulfonamide resistance involves the acquisition of genes encoding enzymes analogous to dihydropteroate synthase (DHPS) (Yun et al., 2013). To date, four sulfonamide-resistance genes exhibiting transferable characteristics have been reported: *sul1*, *sul2*, *sul3*, and *sul4* (Swedberg and Sköld, 1983; Rådström and Swedberg, 1988; Sundström et al., 1988; Perreten and Boerlin, 2003; Razavi et al., 2017). Among the four *sul* genes, *sul1* and *sul2* often form a gene cluster with integron integrase *IntI1* on various plasmids, such as IncQ and pBP1 (Bean et al., 2009; Dungan and Bjorneberg, 2020). The gene cluster is transferred and spread among human and animal pathogens worldwide (Kerrn et al., 2002; Perreten and Boerlin, 2003; Antunes et al., 2005). Among clinically relevant bacteria, *sul3* is of minor importance, but marine unculturable bacteria commonly harbor this gene (Suzuki et al., 2013). It was reported that *sul3* is associated with non-classic class 1 integrons (Vinué et al., 2010). The *sul4* gene was newly discovered in *Chloroflexus* isolated from river sediment (Razavi et al., 2017). Although the host range of *sul4* remains unknown, gene sequences have been deposited in metagenome databases from aquatic environments of Asian and European countries, but no sequences from clinical specimens have been deposited (Razavi et al., 2017). The *sul4* is linked to the transposase IS*CR20*, which may facilitate widespread distribution of the gene through aquatic environments (Razavi et al., 2017). These data indicate that *sul* genes are transferred among clinical and environmental bacteria through various mobile genetic elements (MGEs). This property could lead to the wider spread of *sul* genes.

Horizontal transfer of ARGs in the environment has been reported (Blahna et al., 2006; Aminov, 2011). A variety of MGEs, such as transposons, plasmids, and integrative and conjugative elements, play a role in HGT. Integrons coexisting with MGEs can uptake functional modules from other species and genera (Stokes et al., 2006; Stalder et al., 2012). The *IntI1* gene is an essential part of class 1 integrons and has been reported as a candidate factor for monitoring anthropogenic pollution and ARGs in the environment (Gillings et al., 2015).

Several reports have described the presence of *sul1*, *sul2*, and *sul3* in marine bacteria, predominantly culturable γ-proteobacteria (Wang et al., 2014; Jiang et al., 2019). In the present study, we focused on sulfonamide-resistant marine bacteria, including α- and γ-proteobacteria, which are major bacterial groups in the oceans (Sunagawa et al., 2015). As our results showed that *sul4* is a common sulfonamide-resistance gene, we focused further studies on this gene. The transmissibility of *sul4* and its formation of gene cluster with MGEs have not been reported to date. We conducted a whole-genome analysis using a nanopore long-read sequencing technique to identify the relative positioning of repetitive sequences of ARGs and MGEs in cultured sulfonamide-resistant marine bacteria. This is the first study to detect and determine the cluster structure of *sul4* in marine bacteria.

## 2. Materials and methods

### 2.1. Bacterial strains

Sulfamethoxazole (SMX)-resistant bacteria used in this study were selected from isolates collected from surface seawater off the Takahama coast, Ehime Prefecture, Japan, in May and November, 2015. Ten liters of seawater was sampled each time, then 20 mL was employed for bacterial count experiment. Incubation was performed at 25°C. To select for SMX-resistant bacteria, low-nutrient seawater medium (peptone 0.01 g/L, yeast extract 0.005 g/L, agar 15 g/L in sterile seawater, pH 7.5) and nutrient seawater medium (peptone 5 g/L, yeast extract 1 g/L, agar 15 g/L in sterile seawater, pH 7.5) were used, with the addition of 64 μg/mL of SMX. A total of 74 SMX-resistant isolates (Supplementary Table S1) were obtained.

### 2.2. Bacterial DNA extraction and classification

Total DNA was purified from cultured isolates using a QuickGene DNA Tissue S kit (Kurabo, Osaka). The *16S rRNA* gene sequence (V3 region, 566 bp) was used for bacterial taxonomic identification. PCR mixtures contained 0.025 U of Ex Taq (Takara, Japan), 0.1 μM each of forward and reverse primers, 0.2 mM dNTP mixture (Takara, Japan), 1× Ex Taq buffer, and template DNA (approx. 10 ng). Primers and amplification condition are listed in Supplementary Table S2. PCR products were sequenced on a Genetic Analyzer 3130 (Thermo Fisher Scientific K.K., Japan) with BigDye Terminator and subsequently analyzed using the BLAST program. A sequence identity of ≥97% relative to database species was recognized as indicating the closest species. A phylogenetic tree of the isolates was constructed by Molecular Evolutionary Genetics Analysis 11 (MEGA11, https://www.megasoftware.net/) using the V3 region of the *16S rRNA* gene sequence according to the maximum-likelihood algorithm and Jukes–Cantor model. The phylogenetic tree was visualized using version 5 of Interactive Tree Of Life (iTOL, https://itol.embl.de/) (Letunic and Bork, 2019).

### 2.3. Detection of ARGs and *IntI1*

The *sul1*, *sul2*, *sul3*, *sul4*, *dfrA1* (trimethoprim resistance), and *IntI1* (class 1 integron integrase) among the 74 SMX-resistant isolates were detected using PCR. The *dfrA1* gene plays a role in sulfonamide resistance in a different step of folate synthesis than *sul* (Huovinen et al., 1995). The *IntI1* gene is frequently involved in the transfer of *sul1* and *sul2* (Koczura et al., 2016). The primers and cycle conditions are shown in Supplementary Table S2. PCR was carried out in a mixture of 0.025 U of Ex Taq (Takara, Japan), 0.5 μM each forward and reverse primer, 0.2 mM dNTP mixture (Takara, Japan), 1× Ex Taq buffer, and template DNA (50–100 ng). The amplification products were examined by electrophoresis on a 1.5% agarose gel with GelRed (Nacalai Tesque, Japan) staining. PCR product was confirmed based on band length and sequence.

### 2.4. Genome analysis

Long-read MinION (Oxford Nanopore Technologies, United Kingdom) was used to search for the *sul4* among the genomes of 33 isolates that were *sul4*-positive in PCR screening and to determine the gene structure of the surrounding regions. Total DNA was extracted using a Wizard Genomic DNA Purification kit (Promega, United States) according to the protocol for Gram-negative bacteria. DNA quality was checked using e-spect (Malcom, Japan), and DNA was quantified using a QuantiFluor^®^ dsDNA system (Promega). We prepared the DNA libraries according to the Oxford Nanopore Technologies’ protocol for environmental bacteria. A NEBNext Ultra II End Repair/dA-Tailing Module (New England Biolabs, United States) was used to generate 5′ phosphorylated and 3′ dA-tailed (end-repaired) DNA samples, which were subsequently purified using AMPure XP beads (Beckman Coulter, United Kingdom) and eluted using nuclease-free water. Native Barcoding Expansions (EXP-NBD104 or EXP-NBD114) (Oxford Nanopore Technologies, United Kingdom) were ligated to the end-repaired DNA using Blunt/TA Ligase Master Mix (New England Biolabs). After purification using AMPure XP beads and elution with nuclease-free water, individual barcoded DNA samples were pooled and ligated to sequencing adaptor molecules in Adapter Mix II using NEBNext Quick T4 DNA Ligase (New England Biolabs). The ligation products were washed twice using long fragmentation buffer and eluted with elution buffer. The multiplexed barcoded samples were prepared as sequencing libraries and loaded into a FLO-MIN106D flow cell R9.4.1. Sequencing was carried out on a MinION Mk1B with MinKNOW software (21.10.4) for 24 h using standard settings. Base-calling of FAST5 raw sequencing data was performed using the high-accuracy (*Q*_score_ >9) option with Nanopore Guppy software v.6.0.7 (ONT, United Kingdom). A higher *Q*_score_ may result in reduced detection sensitivity, but it also reduces false positives. Adapter sequences were demultiplexed using Porechop v.0.2.4,^1^ and read quality was checked using Nanoplot with default settings. A summary of the runs is shown in Supplementary Table S3. The reads for each isolate were assembled using Unicycler v.0.4.9 (Wick et al., 2017) with default settings, and the resulting contigs were visualized using Bandage v.0.8.1 (Wick et al., 2017). The gene *sul4* was determined based on amino acid identity relative to deposited sequences in the Comprehensive Antibiotic Resistance Database (CARD) (Jia et al., 2017, https://card.mcmaster.ca/). We searched for high amino acid sequence similarity (*E*-value <1.0, identity >41%, coverage >80%) relative to database-deposited *sul4* gene products (ARO:3004361). MGEs were identified using the reference center for bacterial insertion sequences ISfinder database (ISfinder: https://www-is.biotoul.fr/). All *sul* genes and MGEs were confirmed using tblastn 2.12.0. The threshold values in previous reports were >40 coverage by nucleotide identity (Dai et al., 2022), whereas ours is higher due to longer sequence was targeted. Thus our identification should be reasonable in long-read method.

## 3. Results and discussion

### 3.1. Profile of *sul* genes harbored by SMX-resistant isolates

Occurrence rate of SMX-resistant bacteria on nutrient seawater medium was 27.0% (May) and 8.6% (November), whereas those on low nutrient seawater medium was 39.1% (May) and 97.4% (November). Since marine bacteria generally grow well on low nutrient medium (Carlucci et al., 1986), high rate in low nutrient medium should be reflected by the property.

**Figure 1:**
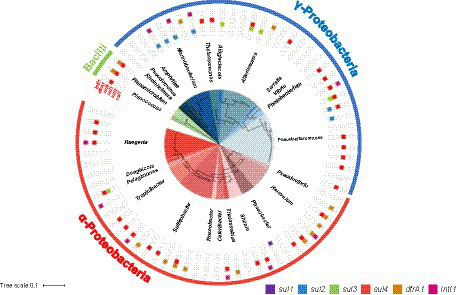
Phylogenetic tree of 74 SMX-resistant isolates based on the *16S rRNA* gene (center circle), and detection of target genes by PCR.

A phylogenetic tree of SMX-resistant isolates based on the *16S rRNA* gene and detection of the *sul1*, *sul2*, *sul3*, *sul4*, *dfrA1*, and *IntI1* genes by PCR are shown in Figure 1. A total of 41 *sul*-positive isolates (55%) were identified among the 74 SMX-resistant isolates. The *sul*-negative isolates should have other mechanisms, such as efflux pumps (Su et al., 2015). Of 37 α-proteobacteria isolates, 16 were *sul4*-positive, 1 was *sul1*-positive, and 1 was *sul3-sul4*-double positive. *dfrA1* was detected in 10 of the 74 SMX-resistant isolates. Three isolates showed the combination *sul4* and *dfrA1*. Of 34 γ-proteobacteria, 14 isolates were *sul4*-positive, 6 were *sul2*-positive, and 4 were *sul3*-positive. Two isolates had *dfrA1* but not *sul* genes. All 3 of the bacilli were *sul4*-positive, and 2 were also *dfrA1*-positive. The frequency of *sul* genes among all 74 SMX-resistant isolates was ranked *sul4* > *sul2* > *sul3* > *sul1*, showing that *sul4* is most common. Most past studies of aquatic bacteria have reported that *sul1* or *sul2* is dominant in cultured bacteria (Rosser and Young, 1999; Stoll et al., 2012; Wang et al., 2014). PCR-based analyses using aquatic environmental DNA also showed that *sul1* or *sul2* is dominant (Muziasari et al., 2014; Suzuki et al., 2019, 2021), which suggests that *sul1* and *sul2* are distributed worldwide among a variety of bacterial communities. On the other hand, Suzuki et al. (2013) detected *sul3* at a higher abundance than *sul1* and *sul2* from a non-culturable community in Manila Bay, Philippines. Taken together with previous reports regarding *sul3*, the high detection rates in the present study suggest that *sul3* and *sul4* might be more common in the marine environment than other environments.

The gene *sul4* was first discovered in river sediments of India in 2017, and it has since been found in environmental metagenomes from several countries (Razavi et al., 2017). However, the host range and distribution are unknown. In the present study, we detected *sul4* by PCR, and the results indicated that *sul4* is dominant among culturable SMX-resistant bacteria from seawater, with an occupation of 45% among *sul*-positive isolates. All *sul4*-positive α-proteobacteria isolates were classified in the family *Rhodobacteraceae* (Figure 1). Bacteria belonging to the *Rhodobacteraceae* are abundant key members of coastal seawater communities that form biofilms (Elifantz et al., 2013). In aquatic environments, biofilms are considered hotspots for horizontal transfer of ARGs (Abe et al., 2020), suggesting there is a high potential for HGT among *sul4*-positive *Rhodobacteraceae* in ocean waters. A ballast water study reported that major ARGs, including *sul1* and *sul2*, were detected in *Rhodobacteraceae* (Lv et al., 2020). In contrast to *sul4*-positive α-proteobacteria, *sul4*-positive γ-proteobacteria were classified into four families (*Alteromonadaceae*, *Vibrionaceae*, *Pseudoalteromonadaceae*, *Colwelliaceae*), suggesting *sul4* has a wide host range in this phylum.

The combination of *sul4* and *IntI1* was found in α-proteobacteria (*Sulfitobacter* sp., *Ruegeria* sp., and *Thalassobius aestuarii*) and γ-proteobacteria (*Photobacterium*), although not at a high frequency (Figure 1). *IntI1* is associated with mobility of ARGs and has been linked to genes conferring resistance to antibiotics and anthropogenic pollutants (Gillings et al., 2015). Some ARGs detected in human and animal pathogens were initially detected in aquatic bacteria (Cabello et al., 2013), suggesting the possibility of transfer of marine-originating ARGs to humans via integrons with *IntI1*. We further examined the primary structure of the peripheral region of *sul4* in the genome to determine whether *sul4* could form a transfer unit.

### 3.2. Genomic analysis of *sul4*-positive isolates

MinION sequencing data for each isolate gave 2,519–45,720 reads, with an average read length of 5,022–24,011 bp and an average read quality of 11–13 (Supplementary Table S3). The genome assembly is summarized in Table 1. After trimming of barcodes and adapters, closed circular contigs were obtained for five isolates via *de novo* assembly, which indicated the genome size was similar to the standard deposited in NCBI. These five isolates were *Celeribacter* sp. (pSMX.B4) and *Ruegeria* sp. (F7CS4) from the α-proteobacteria; and *Alteromonas* sp. (H7CS7), *Pseudoalteromonas* sp. (H7SS12), and *Thalassomonas* sp. (F7CS7) from the γ-proteobacteria (Table 1). Although other isolates gave linear contigs, the total lengths were close to the genome size in the database (Table 1), suggesting that the sequence data covered almost the whole genome. Only *Thalassomonas* sp. F7CS11 showed a small genome size (2,297 kbp). The genomes of related species are reportedly larger than 7 Mbp (Olonade et al., 2015), suggesting that the whole genome sequence was not obtained for F7CS11. However, as we did identify the *sul4*-encoding region in this isolate, it was included for further analysis.

**Table 1:** Genome assemblies of the 23 isolates harboring *sul4*.

Class	Genus	Strain ID	Number of circular contig	Number of liner contig	Total length (kbp)	Largest contig (kbp)	Smallest contig (kbp)
α	*Celeribacter* sp.	p-SMX-B4	4	0	3,986	3,073	8
α	*Phaeobacter* sp.	F0CS4	1	19	5,139	2,933	54
α	*Ruegeria* sp.	H0CS7	1	5	4,527	1,820	110
α	*Ruegeria* sp.	F7CS4	3	0	4,705	4,230	61
α	*Ruegeria* sp.	H7CS9	0	35	8,028	437	66
α	*Shimia* sp.	H7CS3	0	1	4,101	4,101	4,101
γ	*Alteromonas* sp.	H7CS7	2	0	4,705	4,389	161
γ	*Alteromonas* sp.	H7OS11	0	6	4,675	1,742	132
γ	*Photobacterium* sp.	F0CS6	1	1	4,496	3,178	1,318
γ	*Pseudoalteromonas* sp.	H7OS1	0	42	8,434	716	43
γ	*Pseudoalteromonas* sp.	H7OS2	0	16	6,374	2,211	52
γ	*Pseudoalteromonas* sp.	H7OS3	1	10	5,415	1,320	87
γ	*Pseudoalteromonas* sp.	H7OS5	2	2	4,892	3,883	26
γ	*Pseudoalteromonas* sp.	H7OS6	1	2	4,822	3,876	86
γ	*Pseudoalteromonas* sp.	H7OS7	1	2	4,813	3,876	77
γ	*Pseudoalteromonas* sp.	H7OS10	1	13	5,867	3,530	39
γ	*Pseudoalteromonas* sp.	H7SS12	2	0	4,966	3,970	997
γ	*Pseudoalteromonas* sp.	F0CS1	1	1	4,620	3,738	882
γ	*Thalassomonas* sp.	F7CS7	1	0	3,778	3,778	3,778
γ	*Thalassomonas* sp.	F7CS11	0	29	2,297	178	12
B	*Planococcus* sp.	p-SMX-A3	3	1	3,512	3,437	3
B	*Planomicrobium* sp.	p-SMX-A2	0	7	3,301	1,266	106
B	*Planomicrobium* sp.	p-SMX-A4	3	11	4,584	3,397	14

α, α-proteobacteria; γ, γ-proteobacteria; B, bacilli. p-SMX-B4, F0CS4, F7CS7, F7CS4, H7CS7, H7SS12 and p-SMX-A4 showed a complete genome.

Table 2 shows isolates with PCR and tblastn search results for the whole-genome sequence data. Among 33 *sul4*-positives with PCR (Figure 1), 23 isolates were finally confirmed as harboring *sul4* via tblastn search: 6 α-proteobacteria, 14 γ-proteobacteria, and 3 bacilli (Table 2). Sequences of the 23 *sul4*-harboring isolates had hits for sulfonamide-resistance genes in the CARD, which contained efflux pumps, *sul1*, *sul2*, *sul3*, *sul4*, and *dfrA1*. Those genes annotated to *sul4* exhibited amino acid identity of 41%–51% relative to reported *sul4* gene products (ARO:3004361). These data confirmed that the isolates examined in the present study harbor putative *sul4*. In contrast to the above 23 isolates, *sul4* was not detected in the whole genome sequence of the remaining 10 isolates. This false-negative detection may have been due to a high sequencing error rate associated with the nanopore long-read sequencing method. These errors could not be sufficiently corrected, despite our setting of a high *Q*_score_ of >9.

**Table 2:** List of *sul4*-confirmed isolates by nanopore genome sequencing.

Class	Genus	Strain ID	PCR detection						tblastn
			*sul1*	*sul2*	*sul3*	*sul4*	*dfrA1*	*IntI1*	*sul4*
α	*Celeribacter* sp.	p-SMX-B4	−	−	−	+	−	+	1
α	*Phaeobacter* sp.	F0CS4	−	−	−	+	−	−	1
α	*Ruegeria* sp.	H0CS7	−	−	−	+	−	+	1
α	*Ruegeria* sp.	F7CS4	−	−	−	+	−	−	2
α	*Ruegeria* sp.	H7CS9	−	−	−	+	−	−	1
α	*Shimia* sp.	H7CS3	−	−	−	+	−	+	1
γ	*Alteromonas* sp.	H7CS7	−	−	−	+	−	−	1
γ	*Alteromonas* sp.	H7OS11	−	−	+	+	−	−	1
γ	*Photobacterium* sp.	F0CS6	−	−	−	+	−	−	2
γ	*Pseudoalteromonas* sp.	H7OS1	−	−	+	+	−	−	1
γ	*Pseudoalteromonas* sp.	H7OS2	−	−	−	+	−	−	1
γ	*Pseudoalteromonas* sp.	H7OS3	−	−	−	+	−	−	1
γ	*Pseudoalteromonas* sp.	H7OS5	−	−	−	+	−	−	1
γ	*Pseudoalteromonas* sp.	H7OS6	−	−	−	+	−	−	1
γ	*Pseudoalteromonas* sp.	H7OS7	−	−	−	+	−	−	1
γ	*Pseudoalteromonas* sp.	H7OS10	−	−	−	+	−	+	1
γ	*Pseudoalteromonas* sp.	H7SS12	−	+	−	+	−	−	1
γ	*Pseudoalteromonas* sp.	F0CS1	−	−	−	+	−	−	1
γ	*Thalassomonas* sp.	F7CS7	−	−	−	+	−	−	1
γ	*Thalassomonas* sp.	F7CS11	−	−	−	+	−	−	1
B	*Planococcus* sp.	p-SMX-A3	−	−	−	+	−	−	2
B	*Planomicrobium* sp.	p-SMX-A2	−	−	−	+	+	−	2
B	*Planomicrobium* sp.	p-SMX-A4	−	−	−	+	+	−	1

α, α-proteobacteria; γ, γ-proteobacteria; B, bacilli; +, detected with bright positive band; −, no target band. Number in tblastn shows detected copy number of putative *sul4* with high similarity (>41%) to database-deposited *sul4* (A0A2I5YL59-1).

**Figure 2:**
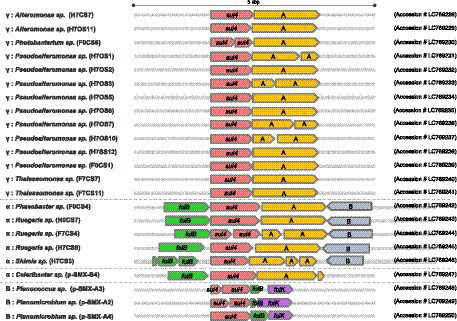
*sul4* and the peripheral region within a 5 kbp area. The gene cluster around *sul4* was classified into 4 types. A: Phosphoglucosamine mutase; B: Permease of the drug/metabolite transporter (DMT) superfamily. Accession number of each cluster is indicated at the right.

Primary structures within the 5-kbp area around *sul4* are shown in Figure 2. Four types of gene arrangement were observed. In type 1, *sul4* was followed by the phosphoglucosamine mutase gene, and this arrangement was observed only in the γ-proteobacteria. In type 2, *sul4* was located immediately after the dihydroneopterin aldolase gene (*folB*), followed by the phosphoglucosamine mutase and permease of the drug/metabolite transporter (DMT) superfamily genes. This type was found in 5 of the α-proteobacteria isolates. Type 3 was similar to type 2; however, the permease DMT superfamily gene was not present. This type was found in α-proteobacteria, *Celeribacter* sp. (pSMX.B4). In type 4, found in bacilli, *sul4* was followed by *folB* and 2-amino-4-hydroxy-6-hydroxymethyldihydropteridine pyrophosphokinase (*folK*). In this manner, the four types of gene arrangement differed among the bacterial classes. Interestingly, none of the types were accompanied by MGEs such as transposons or insertion sequences within the 5-kbp region, suggesting *sul4* is not located on an MGE. *IntI1* was detected in four isolates (Table 2) at a location distant from *sul4*; the distance from *sul4* in p.SMX.B4 and H7CS3 was 1.2 Mbp, and that in H7OS10 was 870 kbp. H0CS7 harbored *IntI1* and *sul4* on different contigs. This distant positioning suggests that *IntI1* is not connected to *sul4.* No other integrase or transposon sequences were found in the *sul4* area (Figure 2), suggesting *sul4* in these marine bacteria might not be a mobilizable gene.

The first report of *sul4* (Razavi et al., 2017) indicated the gene was accompanied by an *attI* site, followed by the complete *qacE* gene, or with a transposase IS*CR20* belonging to the insertion sequence common region (IS*CR*) family. These data suggest *sul4* has been widely distributed via transferable MGEs (Razavi et al., 2017). In contrast, *sul4* was not accompanied by an MGE in the present study, suggesting that marine bacterial *sul4* acquired independently of *Chloroflexus sul4*, which was accompanied by an IS*CR*.

The gene *sul4* in our study showed another interesting characteristic. The peripheral area of *sul4* encodes multiple folate metabolism (*fol*) genes. The *folB* gene was found in types 2, 3, and 4, and *folK* was found in type 4 (Figure 2). The genes encoding the folate synthesis machinery are reportedly clustered on the genome (de Crécy-Lagard et al., 2007), suggesting that *fol* genes around putative *sul4* function in the folate synthesis system. The gene *sul4* possibly encodes a native DHPS in the marine bacteria in our study. The *sul1* and *sul2* genes showed lower *Km* values for *p*-aminobenzoate and higher affinity for sulfonamides than native DHPS (Sköld, 2000). The *sul4* gene product might function as an essential DHPS in the host.

In *Pseudoalteromonas* sp. (H7SS12), *sul2* was found in an area with transposase (Supplementary Figure S1). The *sul2* was suggested to be capable of horizontal transfer between human and animal *Escherichia coli* (Trobos et al., 2009). The gene *sul2* of the isolate H7SS12 is encoded with the aminoglycoside-resistance genes *strB* and *strA* and florfenicol efflux gene *floR* on a transposon area (Supplementary Figure S1), suggesting the presence of a mobilizable ARG-unit with multidrug resistance properties. The resistance to sulfonamide, aminoglycoside, and florfenicol has also been reported in *Vibrio cholerae* (ICDC-VC4210, CDC-VC0143) (Wang et al., 2016) and *V. cholerae* O139 (MO10) (Waldor et al., 1996). The identities of the transposon areas of *Pseudoalteromonas* sp. (H7SS12) relative to these *Vibrio* species were 45% (ICDC-VC4210), 77% (CDC-VC0143), and 68% (MO10) (Supplementary Figure S1) when compared with the complete transposase set. These data suggest that marine bacteria have acquired multidrug resistance via recombination. Furthermore, the concentration site of *strB*, *strA*, and *sul2* was observed on the plasmid pKP27-MCR1 (CP041641.1) of *Klebsiella pneumoniae* (PIMB15ND2KP27) (Mbelle et al., 2019) and on the mobile plasmid of *Salmonella typhimurium* TST207 (Tamamura et al., 2013). This cluster was also found in an Antarctic ice core (1,200 to 2,800 years before present) (Okubo et al., 2019). The multidrug resistance cluster of sulfonamides and aminoglycosides might have been formed via transfer between pathogenic and marine bacteria thousands of years ago.

Some metagenomic studies have reported detection of ARGs in ocean waters (Port et al., 2014; Hatosy and Martiny, 2015; Cuadrat et al., 2020), indicating that efflux channels and tetracycline-resistance genes are abundant (Cuadrat et al., 2020). The results of the present study suggest that metabolic systems of marine bacteria might be capable of playing a role in resistance to antimicrobials, which could then evolute to ARGs that are disseminated to pathogenic bacteria. Marine bacteria could thus be considered an ancestral origin of ARGs.

## 4. Conclusion

Our results showed that *sul4* is present in marine α- and γ-proteobacteria and bacilli without taxonomic bias in the harboring bacteria. This is the first study to detect *sul4* in defined marine bacteria. In addition, *sul4* was not located on an MGEs, suggesting it is not mobile. We found that *sul4* was flanked by genes related to folate synthesis, suggesting that *sul4* codes for a housekeeping DHPS in the host bacteria and is clonally inherited within species. The *sul2* in the *sul4*-harboring isolate was clustered with a transposon, suggesting the presence of a unit for transfer to other bacteria.

## Data availability statement

The original contributions presented in the study are included in the article/Supplementary material, further inquiries can be directed to the corresponding author.

## Author contributions

SaS contributed to conception and design of the study and funding. SuS and AK performed molecular experiments and analysis of data. RK isolated bacteria. KW contributed data curation and manuscript review. SuS and SaS wrote the first draft of the manuscript. All authors contributed to the article and approved the submitted version.

## Funding

This work was supported by KAKENHI 16H01782 and 20H00633, JSPS.

## Conflict of interest

The authors declare that the research was conducted in the absence of any commercial or financial relationships that could be construed as a potential conflict of interest.

## Publisher’s note

All claims expressed in this article are solely those of the authors and do not necessarily represent those of their affiliated organizations, or those of the publisher, the editors and the reviewers. Any product that may be evaluated in this article, or claim that may be made by its manufacturer, is not guaranteed or endorsed by the publisher.

## Footnotes

^1^ https://github.com/rrwick/Porechop
