# Supplementary material for: Marine bacteria harbor the sulfonamide resistance gene sul4 without mobile genetic elements
Source: Front Microbiol. 2023 Sep 14;14:1230548. doi: 10.3389/fmicb.2023.1230548 (PMC10539471; doi:10.3389/fmicb.2023.1230548)
Supplement: Supplementary file 1 [file Data_Sheet_1.PDF]

Table S1. SMX-resistant isolates.

Strain ID	Class	Genus (Accession number)
p-SMX-A3	Bacilli	*Planococcus sp.* (LC768719)
p-SMX-A2	Bacilli	*Planomicrobium sp.* (LC768718)
p-SMX-A4	Bacilli	*Planomicrobium sp.* (LC768720)
H7SS6	α-Proteobacteria	*Aestuariihabitans sp.* (LC768751)
p-SMX-B2	α-Proteobacteria	*Celeribacter sp.* (LC768722)
p-SMX-B4	α-Proteobacteria	*Celeribacter sp.* (LC768723)
p-SMX-E1	α-Proteobacteria	*Halovulum sp.* (LC768732)
p-SMX-E2	α-Proteobacteria	*Halovulum sp.* (LC768733)
p-SMX-E3	α-Proteobacteria	*Halovulum sp.* (LC768734)
p-SMX-E4	α-Proteobacteria	*Halovulum sp.* (LC768735)
p-SMX-E5	α-Proteobacteria	*Halovulum sp.*(LC768736)
H7OS13	α-Proteobacteria	*Pelagimonas sp.* (LC768770)
F0CS4	α-Proteobacteria	*Phaeobacter sp.* (LC768774)
F7CS1	α-Proteobacteria	*Phaeobacter sp.* (LC768782)
F7CS3	α-Proteobacteria	*Phaeobacter sp.* (LC768784)
F0CS8	α-Proteobacteria	*Pseudovibrio sp.* (LC768778)
F0CS7	α-Proteobacteria	*Roseobacter sp.* (LC768777)
H0CS7	α-Proteobacteria	*Ruegeria sp.* (LC768739)
H7CS8	α-Proteobacteria	*Ruegeria sp.* (LC768746)
H7CS9	α-Proteobacteria	*Ruegeria sp.* (LC768747)
F0CS5	α-Proteobacteria	*Ruegeria sp.* (LC768775)
F7CS2	α-Proteobacteria	*Ruegeria sp.* (LC768783)
F7CS4	α-Proteobacteria	*Ruegeria sp.* (LC768785)
H0CS3	α-Proteobacteria	*Ruegeria sp.* (LC768738)
H7CS1	α-Proteobacteria	*Shimia sp.* (LC768740)
H7CS5	α-Proteobacteria	*Shimia sp.* (LC768743)
H7CS3	α-Proteobacteria	*Shimia sp.* (LC768741)
p-SMX-B1	α-Proteobacteria	*Sulfitobacter sp.* (LC768721)
p-SMX-C1	α-Proteobacteria	*Sulfitobacter sp.* (LC768724)
p-SMX-C2	α-Proteobacteria	*Sulfitobacter sp.* (LC768725)
p-SMX-C3	α-Proteobacteria	*Sulfitobacter sp.* (LC768726)
p-SMX-C4	α-Proteobacteria	*Sulfitobacter sp.* (LC768727)
p-SMX-C5	α-Proteobacteria	*Sulfitobacter sp.* (LC768728)
p-SMX-D1	α-Proteobacteria	*Sulfitobacter sp.* (LC768729)
p-SMX-D2	α-Proteobacteria	*Sulfitobacter sp.* (LC768730)
H7CS4	α-Proteobacteria	*Thalassobius sp.* (LC768742)
H7SS3	α-Proteobacteria	*Tropicibacter sp.* (LC768748)
H7SS5	α-Proteobacteria	*Tropicibacter sp.* (LC768750)
H7SS7	α-Proteobacteria	*Tropicibacter sp.* (LC768752)
p-SMX-D4	α-Proteobacteria	*Tropicibacter sp.* (LC768731)
F0CS10	γ-Proteobacteria	*Aliiglaciecola sp.* (LC768780)
H7CS6	γ-Proteobacteria	*Alteromonas sp.* (LC768744)
H7CS7	γ-Proteobacteria	*Alteromonas sp.* (LC768745)
F7CS6	γ-Proteobacteria	*Alteromonas sp.* (LC768787)
F7CS9	γ-Proteobacteria	*Alteromonas sp.* (LC768789)
H7OS4	γ-Proteobacteria	*Alteromonas sp.* (LC768762)
H7OS11	γ-Proteobacteria	*Alteromonas sp.* (LC768769)
F7CS5	γ-Proteobacteria	*Alteromonas sp.* (LC768786)
F7CS10	γ-Proteobacteria	*Alteromonas sp.* (LC768790)
H0CS2	γ-Proteobacteria	*Amphritea sp.* (LC768737)
H7SS8	γ-Proteobacteria	*Marinobacterium sp.* (LC768753)
H7SS9	γ-Proteobacteria	*Marinobacterium sp.* (LC768754)
H7SS10	γ-Proteobacteria	*Marinobacterium sp.* (LC768755)
H7SS11	γ-Proteobacteria	*Marinobacterium sp.* (LC768756)
H7SS13	γ-Proteobacteria	*Marinobacterium sp.* (LC768758)
F0CS6	γ-Proteobacteria	*Photobacterium sp.* (LC768776)
H7OS1	γ-Proteobacteria	*Pseudoalteromonas sp.* (LC768759)
H7OS10	γ-Proteobacteria	*Pseudoalteromonas sp.* (LC768768)
H7SS4	γ-Proteobacteria	*Pseudoalteromonas sp.* (LC768749)
H7SS12	γ-Proteobacteria	*Pseudoalteromonas sp.* (LC768757)
F0CS1	γ-Proteobacteria	*Pseudoalteromonas sp.* (LC768771)
H7OS2	γ-Proteobacteria	*Pseudoalteromonas sp.* (LC768760)
H7OS3	γ-Proteobacteria	*Pseudoalteromonas sp.* (LC768761)
H7OS5	γ-Proteobacteria	Pseudoalteromonas sp. (LC768763)
H7OS6	γ-Proteobacteria	*Pseudoalteromonas sp.* (LC768764)
H7OS7	γ-Proteobacteria	*Pseudoalteromonas sp.* (LC768765)
H7OS8	γ-Proteobacteria	*Pseudoalteromonas sp.* (LC768766)
H7OS9	γ-Proteobacteria	*Pseudoalteromonas sp.* (LC768767)
F0CS3	γ-Proteobacteria	*Pseudomonas sp.* (LC768773)
F0CS11	γ-Proteobacteria	*Rheinheimera sp.* (LC768781)
F0CS2	γ-Proteobacteria	*Serratia sp.* (LC768772)
F7CS7	γ-Proteobacteria	*Thalassomonas sp.* (LC768788)
F7CS11	γ-Proteobacteria	*Thalassomonas sp.* (LC768791)
F0CS9	γ-Proteobacteria	*Vibrio sp.* (LC768779)

Table S2. Primers and amplification conditions for PCR.

Primer name	Primer sequence (5' -> 3')	PCR cycle condition	Product size (bp)	Target gene	Reference
341f	CCTACGGGAGGCAGCAG	Denaturation at 94℃ for 5 min, followed by 25 cycles : denaturing at 94℃ for 60 sec, annealing at 58 ℃ for 60 sec, extension at 72℃ for 60 sec ; and final extension at 72 ℃ for 7 min	566	16SrRNA	Muyzer et al.(1993)
907r	CCGTCAATTCMTTTGAGTTT				Lane et al.(1985)
sul1F	CTGAACGATATCCAAGGATTYCC	Denaturation at 94℃ for 2 min, followed by 35 cycles : denaturing at 98℃ for 10 sec, annealing at 58 ℃ for 30 sec, extension at 72℃ for 1 min ; and final extension at 68 ℃ for 10 min	245	sul1	Heuer and Smalla (2007)
sul1R	AAAAATCCCATCCCCGGRTC				
sul2F	CTCAATGATATTCGCGGTTTYCC			sul2	
sul2R	AAAAACCCCATGCCGGGRTC				
sul3F	ATTAATGATATTCAAGGTTTYCC			sul3	
sul3R	AAGAAGCCCATACCCGGRTC				
sul4_L2F	ATGTCAACCACACTAACCAGCTT	Denaturation at 94℃ for 5 min, followed by 35 cycles : denaturing at 94℃ for 30 sec, annealing at 62 ℃ for 60 sec, extension at 72℃ for 45 sec ; and final extension at 72 ℃ for 5 min	858	sul4	This study
sul4_L3R	AGCACTGAAATCCTTTAACGTCTC				
dfrA1−1f	TGGTAGCTATATCGAAGAATGGAGT	Denaturation at 94℃ for 5 min, followed by 35 cycles : denaturing at 94℃ for 30 sec, annealing at 58 ℃ for 30 sec, extension at 72℃ for 1 min ; and final extension at 72℃ for 5 min	425	dfrA1	Shah et al. (2014)
dfrA1-1R	TATGTTAGAGGCGAAGTCTTGGGTA				
HS463a	CTGGATTTCGATCACGGCACG	Denaturation at 94℃ for 5 min, followed by 30 cycles : denaturing at 94℃ for 30 sec, annealing at 64 ℃ for 30 sec, extension at 72℃ for 30 sec ; and final extension at 72 ℃ for 5 min	473	IntI1	Waldron and Gillings (2015)
HS464	ACATGCGTGTAAATCATCGTCG				

Table S3. Summary of sequencing results of five runs.

Class	Genus	Strain ID	Number of reads	Total bases (Mb)	Mean read length (bp)	Mean read quality	Median read length （bp）
α	*Celeribacter sp.*	p-SMX-B4	8,422	106	12,601	12	8,087
α	*Phaeobacter sp.*	F0CS4	31,188	625	20,055	13	16,722
α	*Ruegeria sp.*	H0CS7	4,796	64	13,446	12	7,552
α	*Ruegeria sp.*	F7CS4	6,218	109	17,528	12	12,323
α	*Ruegeria sp.*	H7CS9	9,758	134	13,750	12	8,013
α	*Shimia sp.*	H7CS3	6,330	73	11,583	12	5,700
γ	*Alteromonas sp.*	H7CS7	35,186	285	8,094	12	4,052
γ	*Alteromonas sp.*	H7OS11	8,252	97	11,699	13	7,375
γ	*Photobacterium sp.*	F0CS6	11,412	143	12,554	12	8,289
γ	*Pseudoalteromonas sp.*	H7OS1	45,720	385	8,425	13	3,743
γ	*Pseudoalteromonas sp.*	H7OS2	36,490	624	17,114	13	13,244
γ	*Pseudoalteromonas sp.*	H7OS3	42,766	677	15,837	13	11,351
γ	*Pseudoalteromonas sp.*	H7OS5	22,933	526	22,937	13	20,160
γ	*Pseudoalteromonas sp.*	H7OS6	9,108	186	20,385	13	17,514
γ	*Pseudoalteromonas sp.*	H7OS7	4,681	112	24,011	13	20,427
γ	*Pseudoalteromonas sp.*	H7OS10	33,101	651	19,682	13	17,022
γ	*Pseudoalteromonas sp.*	H7SS12	41,199	319	7,750	12	4,762
γ	*Pseudoalteromonas sp.*	F0CS1	12,641	99	7,793	12	3,057
γ	*Thalassomonas sp.*	F7CS7	5,584	98	17,557	13	11,619
γ	*Thalassomonas sp.*	F7CS11	8,871	45	5,022	11	2,878
B	*Planococcus sp.*	p-SMX-A3	8,382	91	10,909	11	5,713
B	*Planomicrobium sp.*	p-SMX-A2	2,519	39	15,485	11	9,468
B	*Planomicrobium sp.*	p-SMX-A4	15,303	349	22,810	13	18,581

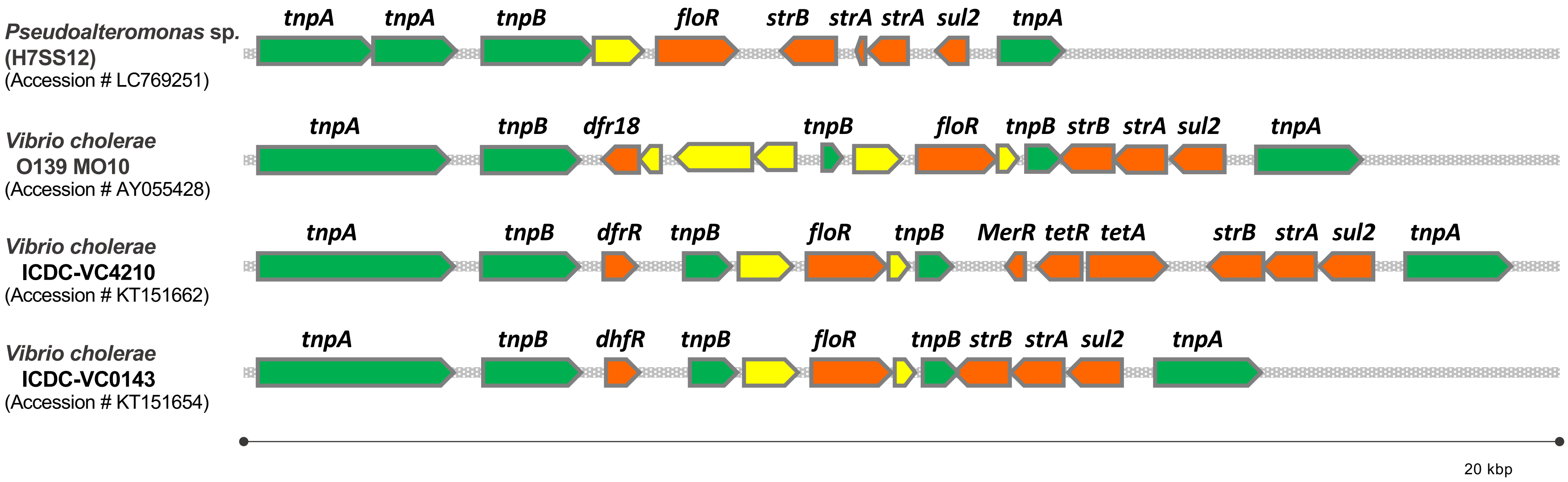

Fig. S1. Cluster of *sul2* with other ARGs in *Pseudoalteromonas* sp. H7SS12. Reference sequences are *Vibrio cholerae* O139 MO10 (Waldor et al., 1996), *V. cholerae* ICDC-VC4210 (Wang et al., 2016) and ICDC-VC0143 (Wang et al., 2016). Colors show function. Green, transposition; orange, resistance; yellow, others.
